# A Comparison of Laparoscopies and Laparotomies for Radical Hysterectomy in Stage IA1-IB1 Cervical Cancer Patients: A Single Team With 18 Years of Experience

**DOI:** 10.3389/fonc.2020.01738

**Published:** 2020-08-28

**Authors:** Meng Qin, Li Siyi, Hui-Fang Huang, Yan Li, Yu Gu, Wei Wang, Ying Shan, Jie Yin, Yong-Xue Wang, Yan Cai, Jia-Yu Chen, Ying Jin, Ling-Ya Pan

**Affiliations:** Department of Obstetrics and Gynecology, Peking Union Medical College Hospital, Chinese Academy of Medical Sciences and Peking Union Medical College, Beijing, China

**Keywords:** cervical cancer, laparoscopy, radical hysterectomy, survival outcome, learning curve, single team

## Abstract

**Objective:**

To investigate the safety and efficacy of abdominal radical hysterectomy (ARH) and laparoscopic radical hysterectomy (LRH) in managing early-stage cervical cancer.

**Methods:**

This retrospective study comprised patients with FIGO stage IA1 with lymphovascular space invasion (LVSI), IA2, and IB1 cervical cancer who underwent radical hysterectomy performed by a single gynecologic oncology team at Peking Union Medical College Hospital from 2000–2018. The clinicopathological characteristics, surgical outcomes, and survival outcomes were compared between the two groups.

**Results:**

The ARH and LRH groups consisted of 84 and 172 patients, respectively. The 5-year progression-free survival (PFS) rates were 89.3 and 95.9% in the ARH and LRH groups (*P* = 0.122, adjusted HR = 0.449, 95% CI: 0.162–1.239), respectively, while the 5-year overall survival (OS) rates were 95.2 and 98.8%, respectively (*P* = 0.578, adjusted HR = 0.650, 95% CI: 0.143–2.961). The presence of more than two comorbidities led to poor OS (*P* = 0.011). For patients with a BMI greater than 24 kg/m^2^, LRH was associated with better PFS (*P* = 0.039). Compared with ARH, LRH was associated with a shorter operation time (248.8 vs. 176.9 min, *P* < 0.001), less blood loss (670.2 vs. 200.9 ml, *P* < 0.001), and lower postoperative ileus rates (2.4% vs. 0%, *P* = 0.042). No significant differences were observed in PFS and OS between 2006–2012, 2013–2015, and 2016–2018 in the LRH group (*P* = 0.126 and *P* = 0.583).

**Conclusion:**

Compared with ARH, LRH yields similar survival and improved surgical outcomes in patients with early-stage cervical cancer. LRH is not inferior to ARH for select cervical cancer patients treated by a single team with adequate laparoscopy experience.

## Introduction

Cervical cancer is one of the most common cancers among women and ranks fourth globally in both incidence and mortality, with 570,000 cases and 311,000 deaths in 2018 (1). Although the incidence and mortality rates of cervical cancer have reportedly been decreasing worldwide, a non-negligible burden of cervical cancer remains in developing countries (2). In China, newly diagnosed cases account for 12% of new cases worldwide and contribute to a progressive increase among young women, probably due to the popularization of screening and a greater incidence of human papillomavirus infections (3, 4).

Patients are increasingly diagnosed with cervical cancer at an early stage in China, and most are treated by surgery (5). Radical hysterectomy (RH) with bilateral pelvic lymph node dissection has become the standard treatment for early-stage cervical cancer, that is, stage IA1 with lymphovascular space invasion (LVSI)-IB1 disease, as defined by the International Federation of Gynecology and Obstetrics (FIGO) staging system in 2009 (6). For patients with locally advanced disease with a bulk lesion >4 cm (FIGO stage IB2 or IIA2), radical surgery accompanied by adjuvant therapy is also a curative treatment modality (7, 8).

Abdominal radical hysterectomy (ARH) has been the traditional approach to manage early-stage patients for many years. The utilization of laparoscopy for minimally invasive surgery (MIS) is a growing trend in gynecologic oncology (9). Laparoscopic radical hysterectomy (LRH) was shown to have more favorable short-term outcomes than open surgery, including less blood loss, lower transfusion rates, shorter operative times and hospital stays, and fewer postoperative complications (10–14). Several retrospective studies also showed that LRH and ARH have equivalent progression-free survival (PFS) and overall survival (OS) rates (11, 15, 16). LRH has been gradually accepted as a reasonable alternative to ARH for patients with early-stage cervical cancer. However, recent studies have reached the opposite conclusion – that LRH is associated with poor long-term outcomes (17–20). As the only prospective randomized trial, the Laparoscopic Approach to Carcinoma of the Cervix (LACC) trial may provide high-quality evidence to support the notion that MIS, including laparoscopic and robot-assisted RH, leads to higher rates of recurrence and lower rates of OS than open surgery (19). These unexpected results from new research motivated us to reassess the safety and effectiveness of MIS techniques for RH in the management of patients with early-stage cervical cancer treated by a single team.

Currently, the survival benefits of LRH vs. those of ARH continue to be controversial. Thus, this retrospective study obtained clinical data from our team at a single medical center in China and compared the oncological outcomes between LRH and ARH in cases of stage IA1 with LVSI to IB1 cervical cancer.

## Materials and Methods

### Study Population

This retrospective study included all patients with FIGO stage IA1 with LVSI, IA2, and IB1 cervical cancer (6) who received type B or C RH [according to the Querleu and Morrow classification (21)] as the primary treatment by a single gynecologic oncology team at Peking Union Medical College Hospital (PUMCH) from January 1, 2000 to March 30, 2018. Patients with any of the following characteristics were excluded: (1) received neoadjuvant chemotherapy (NACT) or neoadjuvant radiotherapy; (2) had rare pathological types other than squamous cell carcinoma (SCC), adenocarcinoma, or adenosquamous carcinoma; and (3) underwent fertility-sparing surgery (such as trachelectomy) or incomplete pelvic lymphadenectomy. All patients provided written informed consent under the approval of the Ethics Committee of PUMCH (Project ID: CIFMS-2017-I2M-1-002). All ethical standards including ethics committee approval and the consent procedure were in accordance with international conventions.

### Data Collection

For this retrospective analysis, the following data were extracted from the medical records from the Hospital Information System and through telephone interviews: patient information, clinicopathological characteristics, adjuvant treatment, risk factors, surgical outcomes, survival outcomes, and sites of recurrence. The risk factors included positive resection margin, parametrial involvement, lymph node metastasis, the depth of cervical stromal invasion, and tumor mass size. The surgical outcomes included the length of operation time, volume of intraoperative blood loss, presence of blood transfusion, length of hospital stay (LOS), and postoperative complications. Intraoperative blood loss was assessed through measuring the weight of intraoperative gauzes, suction canisters or visual assessment of experienced attending surgeons. A standard small swab that was almost completely saturated with blood represented an estimated blood loss of approximately 30 ml. The amount of blood loss during LRH by suction canisters was approximately the amount of suction fluid in the suction bottle minus the amount of water flushed. The postoperative complications included infections, ureteral fistula, urinary dysfunction, ileus, lymphocele, thrombus, and so on. The type of postoperative ileus was mainly mechanical ileus, which was defined as the form of ileus lasting more than 3 days after surgery due to adhesion, inflammation or anesthesia. The postoperative ileus was diagnosed by the principal symptoms (abdominal pain, distention, constipation, or vomiting, etc.) and findings on an X-ray suggestive of ileus, which include multiple air-fluid levels throughout the abdomen and elevated diaphragm with dilatation of both the large and small intestines. The length of hospital stay was defined as the interval time between the date of admission and the date of discharge to hospitalization. The survival outcomes of PFS and OS were the most important results for this study. PFS was defined as the time interval between the date of the first diagnosis and the date of cervical cancer progression. OS was defined as the time interval between the date of the first diagnosis and the date of death (22). Patients who met the study inclusion criteria were divided into two groups: the ARH group, whose patients underwent RH by laparotomy, and the LRH group, whose patients underwent RH by laparoscopy.

### Subgroup Analysis

We selected six different stratifying variables, which can be obtained before the surgery, for the subgroup analysis: age (age <50 years; age ≥50 years), body mass index (BMI <24 kg/m^2^; BMI ≥24 kg/m^2^; BMI ≥28 kg/m^2^), comorbidities (0; 1; ≥2), FIGO stage (IA1 with LVSI + IA2; IB1), histology (SCC; non-SCC), and tumor mass (size ≤2 cm; size >2 cm) (19). In addition, the years in which LRH was performed were compared to evaluate the long-term laparoscopic skill of our team in this study.

### Statistical Analysis

All statistical analyses were performed using SPSS software (version 23.0; SPSS Inc., Chicago, IL, United States) and graphs were generated using GraphPad Prism software for Macbook (version 7.0; GraphPad software Inc., San Diego, CA, United States). Student’s *t*-tests and Mann–Whitney U tests were used to compare continuous variables. Pearson’s Chi-squared tests and Fisher’s exact tests were used to compare categorical variables (23, 24). The survival analysis was performed using Kaplan–Meier curves and the log-rank test. Each of the factors related to survival outcomes was individually evaluated using a Cox regression model in a univariate analysis. Then, all the variables with *P-*values <0.200 and meaningful variables based on the univariate analysis were calculated by the Cox proportional hazards regression model in a multivariate analysis. The association was evaluated by hazard ratios (HRs) and 95% confidence intervals (CIs). All statistical significance was set at *P* < 0.050.

## Results

### Comparison of the Clinical and Operative Pathological Characteristics Between the ARH and LRH Groups

Table 1 shows the clinical and pathological characteristics of cervical cancer patients in the ARH and LRH groups. Overall, 256 cervical cancer patients were included in this retrospective analysis. The ARH group contained 84 patients, while the LRH group contained 172 patients. The median follow-up time was 59 months. The mean ages were 42.8 years in the ARH group and 44.3 years in the LRH group, slightly younger than the patients of the LACC trial (46 years). No significant differences were observed in any of the included clinical characteristics between the two groups, including age (*P* = 0.247), BMI (*P* = 0.615), comorbidities (*P* = 0.342), and FIGO stage (*P* = 0.360). The rates of overall postoperative adjuvant treatment were similar between the two groups (*P* = 0.293): 44.0% in the ARH group and 37.2% in the LRH group. Furthermore, no significant differences were found between two groups in the rates of adjuvant chemotherapy only (*P* = 0.170), adjuvant radiotherapy only (*P* = 0.689), and concurrent chemoradiation therapy (CCRT, *P* = 0.051). In terms of the postoperative pathology characteristics, no significant differences were observed in histology (*P* = 0.080), tumor grade (*P* = 0.072), or any of the risk factors. Therefore, all variables were equally comparable for the survival analysis.

**TABLE 1:** The clinical and pathological characteristics of patients in ARH and LRH groups.

Characteristics	Total (*N* = 256)	ARH group (*n* = 84)	LRH group (*n* = 172)	*P*
**Clinical characteristics**				
Age (mean + SD)	43.8(±8.3)	42.8(±8.3)	44.3(±8.2)	0.247
<50 year	196 (76.6)	68 (81.0)	128 (74.4)	
≥50 year	60 (23.4)	16 (19.0)	44 (25.6)	
BMI (kg/m^2^)	23.1(±2.9)	23.2(±3.0)	23.1(±2.8)	0.615
<24.0	164 (64.1)	52 (61.9)	112 (65.1)	
≥24.0	92 (35.9)	32 (38.1)	60 (34.9)	
≥24.0, < 28	77 (30.0)	28 (33.3)	49 (28.5)	
≥28	15 (5.9)	4 (4.8)	11 (6.4)	
≥30	3 (1.2)	1 (1.2)	2 (1.2)	
**Comorbidities**				0.342
0	213 (83.2)	74 (88.1)	139 (80.8)	
1	34 (13.3)	8 (9.5)	26 (15.1)	
≥2	9 (3.5)	2 (2.4)	7 (4.1)	
**FIGO stage**				0.360
IA1 with positive LVSI	4 (1.6)	1 (1.2)	3 (1.7)	
IA2	17 (6.6)	3 (3.5)	14 (8.1)	
IB1	235 (91.8)	80 (95.3)	155 (90.2)	
**Adjuvant treatment**				
Yes	101 (39.5)	37 (44.0)	64 (37.2)	0.293
Chemotherapy only	13 (5.1)	2 (2.4)	11 (6.4)	0.170
Radiotherapy only	11 (4.3)	3 (3.6)	8 (4.7)	0.689
CCRT	77 (30.1)	32 (38.1)	45 (26.2)	0.051
**Pathological characteristics**				
Histology				0.080
Squamous-cell carcinoma	204 (79.7)	72 (85.7)	132 (76.8)	
Adenocarcinoma	43 (16.8)	8 (9.5)	35 (20.3)	
Adenosquamous carcinoma	9 (3.5)	4 (4.8)	5 (2.9)	
**Tumor grade**				
G1	133 (52.0)	36 (42.9)	97 (56.4)	0.072
G2	66 (25.8)	23 (27.4)	43 (25.0)	
G3	57 (22.2)	25 (29.7)	32 (18.6)	
**Risk factors**				
Positive resection margin	1 (0.4)	0 (0.0)	1 (0.1)	0.484
Parametrial involvement	6 (2.3)	1 (1.2)	5 (2.9)	0.394
Lymph node metastasis	24 (9.4)	9 (10.7)	15 (8.7)	0.607
**Cervical stromal invasion depth**				0.410
<1/3	152 (59.4)	45 (53.6)	107 (62.2)	
≥1/3, <2/3	81 (31.6)	30 (35.7)	51 (29.7)	
≥2/3	23 (9.0)	9 (10.7)	14 (8.1)	
**Tumor mass size**				0.282
≤2 cm	175 (68.4)	54 (64.3)	121 (70.3)	
>2 cm	81 (31.6)	30 (35.7)	51 (29.7)	

*Data are presented as number (%) or mean (±SD) or median (±IQR). ARH, abdominal radical hysterectomy; LRH, laparoscopic radical hysterectomy; BMI, body mass index; CCRT, concurrent chemoradiation therapy.*

### Comparison of the Surgical Outcomes and Recurrence Sites Between the ARH and LRH Groups

The upper part of Table 2 shows the short-term surgical outcomes in the ARH and LRH groups. The operation time of the ARH group was significantly longer than that of the LRH group (248.8 vs. 176.9 min, *P* < 0.001). The volume of blood loss during surgery in the ARH group was also greater than that in the LRH group (670.2 vs. 200.9 ml, *P* < 0.001). More cases in the ARH group than in the LRH group required a blood transfusion (21 vs. 3, *P* < 0.001). No significant difference was seen in the cases with postoperative complications (*P* = 0.566), except that the LRH group had a lower incidence of postoperative ileus (2.4% vs. 0%, *P* = 0.042). One reason may be that the intestines are easily moved during laparotomy; thus, the high rate of adhesion led to postoperative ileus. In this study, although no significant difference of LOS was observed between the two surgery groups (*P* = 0.057), the average LOS in the ARH group was longer than that of the LRH group (15.9 vs. 14.5 days). The reason why no difference was found in LOS may be the different styles of hospitalization management between Chinese hospitals and western hospitals. Cervical cancer is covered by the National Critical illness insurance in China. The patient’s surgical and hospital expenses can be almost entirely reimbursed. At PUMCH, all cervical cancer patients who plan to receive RH are admitted to the hospital 3 days before surgery. Then, the patient is not discharged from the hospital until the drainage tube and urine tube are removed. Therefore, the average LOS was slightly longer. However, considering the shorter operative time and lower amount of operative blood loss, the benefits of surgery and hospitalization when using LRH for cervical cancer were remarkable.

**TABLE 2:** The surgical and survival outcomes of patients in ARH and LRH groups.

Characteristics	Total (*N* = 256)	ARH group (*n* = 84)	LRH group (*n* = 172)	*P*
Length of operation time (minute)	200.5(±54.5)	248.8(±50.9)	176.9(±38.3)	< 0.001
Volume of intraoperative blood loss (ml)	354.9(±379.5)	670.2(±512.0)	200.9(±121.5)	< 0.001
The presence of blood transfusion	24 (9.4)	21 (25.0)	3 (1.7)	< 0.001
Length of hospital stay (day)	14.9(±6.4)	15.9(±8.0)	14.5(±5.5)	0.057
**Postoperative complications**				
No	218 (85.2)	70 (83.3)	148 (86.0)	0.566
Infections	14 (6.4)	4 (4.8)	10 (5.8)	0.728
Ureteral fistula	4 (1.6)	1 (1.2)	3 (1.7)	0.737
Urinary dysfunction	9 (3.5)	1 (1.2)	8 (4.7)	0.158
Ileus	2 (0.8)	2 (2.4)	0 (0)	0.042
Lymphocele	9 (3.5)	4 (4.8)	5 (2.9)	0.449
Thrombus	4 (1.6)	1 (1.2)	3 (1.7)	0.737
Stay in ICU	2 (0.8)	1 (1.2)	1 (0.6)	0.603
No recurrence	239 (93.4)	75 (89.3)	164 (95.4)	0.067
Recurrent death	9 (3.5)	5 (5.9)	4 (2.3)	0.139
Recurrent but alive	8 (3.1)	4 (4.8)	4 (2.3)	0.293
**Sites of recurrence**				0.549
Inside of pelvis	7 (41.2)	4 (44.4)	3 (37.5)	
Outside of pelvis	9 (52.9)	5 (55.6)	4 (50.0)	
Inside and outside of pelvis	1 (5.9)	0 (0)	1 (12.5)	

*Data are presented as number (%) or mean (±SD) or median (±IQR). ARH, abdominal radical hysterectomy; LRH, laparoscopic radical hysterectomy; ICU, Intensive care unit.*

The lower part of Table 2 shows the long-term survival outcomes between the ARH and LRH groups. The ARH group contained 9 (10.7%) recurrent patients and 5 (6.0%) deceased patients compared with 8 (4.7%) recurrent patients and 4 (2.3%) deceased patients in the LRH group. In this study, 17 of the included patients had cervical cancer recurrence, and among them 9 patients died. All deceased patients had cancer-related deaths. Among all recurrent cases in the ARH group, the recurrences of 4 (44.4%) patients occurred inside the pelvis, those of 5 (55.6%) patients occurred outside the pelvis, which including metastases to the lung and bone. No patients experienced recurrences both inside and outside the pelvis in the ARH group. In contrast, the number (proportions) of patients in the LRH group with recurrences inside the pelvis, outside the pelvis, and both inside and outside the pelvis were 3 (37.5%), 4 (50.0%), and 1 (12.5%), respectively. For the four patients with recurrences outside the pelvis, two occurred in the lung, one occurred in the bone, and one occurred in the liver surface. Meanwhile, the one patient with recurrences both inside and outside the pelvis in the LRH group had metastasis in the rectum, liver surface, and peritoneum after operation. However, no difference was observed in the sites of recurrence between the two groups (*P* = 0.549).

### Comparison of the Survival Outcomes Between the ARH and LRH Groups

Tables 3, 4 respectively show the results of the univariate and multivariate analyses of PFS and OS. In the univariate analysis of PFS, the *P-*values of six factors were less than 0.200: BMI (*P* = 0.056), comorbidities (*P* = 0.196), parametrial involvement (*P* = 0.161), the depth of cervical stromal invasion (*P* = 0.144), tumor size (*P* = 0.017), and operation approach (*P* = 0.144). In the univariate analysis of OS, the *P-*value of comorbidities (*P* = 0.019) and adjuvant treatment (*P* = 0.137) was less than 0.200, and the operation approach (*P* = 0.780) should have been considered a meaningful variable. After the multivariate analysis, no factors were associated with PFS. However, the presence of more than two comorbidities led to poor OS (*P* = 0.011, adjusted HR = 14.230, 95% CI: 2.463–82.206). Ten of the included patients had more than two comorbidities, mainly hypertension (5/10), diabetes (3/10), and other cardiovascular or cerebrovascular diseases. Among them, two patients (2/10) experienced recurrence and died. The reason may be that the patients with multiple comorbidities had a weak performance status, poor body tolerance or poor treatment compliance.

**TABLE 3:** The univariate analysis of factors associated with PFS and OS in overall included patients.

Variables	*N*	Progression-free survival			Overall survival		
		HR	95% CI	*P*	HR	95% CI	*P*
**Age**				0.563			0.418
<50 year	196	1			1		
≥50 year	60	1.361	0.479–3.863		1.774	0.443–7.099	
**BMI**				0.056			0.283
<24 kg/m^2^	164	1			1		
≥24 kg/m^2^	92	2.567	0.977–6.747		2.062	0.550–7.731	
**Comorbidities**				0.196			0.019
0	213	1			1		
1	34	1.005	0.227–4.456		1.177	0.141–9.805	
≥2	9	3.904	0.878–17.361		10.438	2.013–54.113	
**FIGO stage**				0.406			0.577
IA1 with LVSI + IA2	21	1			1		
IB1	235	23.102	0.014–38018.254		22.675	3.9 × 10^–4^–1318638.17	
**Histology**				0.830			0.689
SCC	204	1			1		
Non-SCC	52	0.873	0.250–3.040		0.653	0.081–5.250	
**Tumor grade**				0.900			0.705
G1	133	1			1		
G2	66	1.297	0.424–3.967		1.790	0.357–8.983	
G3	57	1.133	0.341–3.767		1.846	0.367–9.286	
**Adjuvant treatment**				0.224			0.137
No	155	1			1		
Yes	101	1.807	0.697–4.684		2.879	0.715–11.593	
**Positive resection margin**				0.858			0.896
No	255	1			1		
Yes	1	0.049	2.7 × 10^–12^–9.1 × 10^12^		0.049	1.3 × 10^–21^–1.9 × 10^18^	
**Parametrial involvement**				0.161			0.819
No	250	1			1		
Yes	6	4.271	0.560–32.599		0.049	2.000–8.605 × 10^–9^	
**Lymph node metastasis**				0.212			0.878
No	232	1			1		
Yes	24	2.211	0.635–7.698		1.176	0.147–9.147	
**Cervical stromal invasion depth**				0.144			0.489
<1/3	152	1			1		
≥1/3, <2/3	81	2.425	0.841–6.990		1.284	0.287–5.740	
≥2/3	23	3.314	0.829–13.254		2.807	0.512–15.387	
**Tumor mass size**				0.017			0.495
≤2 cm	175	1			1		
>2 cm	81	3.259	1.240–8.562		1.584	0.422–5.941	
**Operation approach**				0.144			0.780
ARH	84	1			1		
LRH	172	0.493	0.187–1.296		0.816	0.196–3.403	

*ARH, abdominal radical hysterectomy; LRH, laparoscopic radical hysterectomy; BMI, body mass index; FIGO, International Federation of Gynecology and Obstetrics; SCC, squamous-cell carcinoma.*

**TABLE 4:** The multivariate analysis of factors associated with PFS and OS in overall included patients.

Variables	*N*	Progression-free survival			Overall survival		
		HR	95% CI	*P*	HR	95% CI	*P*
**BMI**				0.071			
<24 kg/m^2^	164	1					
≥24 kg/m^2^	92	2.538	0.924–6.968				
**Comorbidities**				0.174			0.011
0	213	1			1		
1	34	0.931	0.207–4.193		1.174	0.139–9.886	
≥2	9	4.614	0.901–23.624		14.230	2.463–82.206	
**Adjuvant treatment**							0.097
No	155				1		
Yes	101				3.333	0.806–13.794	
**Parametrial involvement**				0.220			
No	250	1					
Yes	6	3.852	0.446–33.259				
**Cervical stromal invasion depth**				0.508			
<1/3	152	1					
≥1/3, <2/3	81	1.945	0.635–5.956				
≥2/3	23	1.540	0.336–7.069				
**Tumor mass size**				0.085			
≤2 cm	175	1					
>2 cm	81	2.605	0.877–7.731				
**Operation approach**							
ARH	84	1		0.122	1		0.578
LRH	172	0.449	0.162–1.239		0.650	0.143–2.961	

*ARH, abdominal radical hysterectomy; LRH, laparoscopic radical hysterectomy; BMI, body mass index.*

**FIGURE 1:**
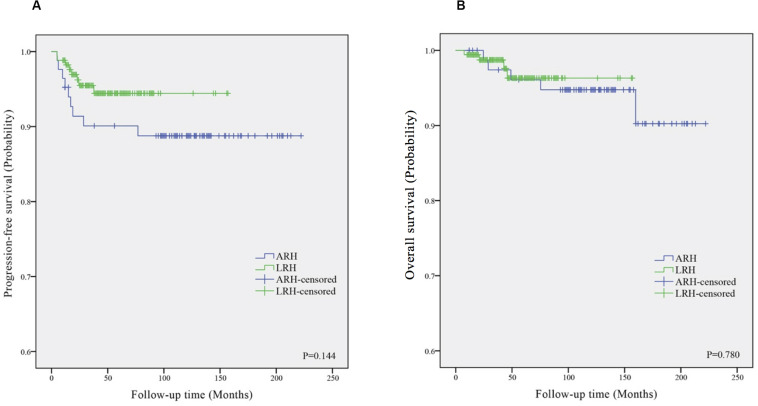
The Kaplan–Meier survival curves of progression-free survival **(A)** and overall survival **(B)** in ARH and LRH groups. ARH, abdominal radical hysterectomy; LRH, laparoscopic radical hysterectomy.

Figure 1 shows the Kaplan–Meier survival curves of the PFS (Figure 1A) and OS (Figure 1B) in the ARH and LRH groups, which were measured from the data of all the patients included in our study. The ARH group demonstrated a worse trend in PFS rates (3-year rate, 89.3% vs. 97.1%; 5-year rate, 89.3% vs. 95.9%) as well as in OS rates (3-year rate, 95.2% vs. 98.8%; 5-year rate, 95.2% vs. 98.8%) than the LRH group. However, no significant differences were found in the PFS (*P* = 0.144) and OS (*P* = 0.780) in the univariate analysis or in the multivariate analysis (*P* = 0.122, adjusted HR = 0.449, 95% CI: 0.162–1.239; *P* = 0.578, adjusted HR = 0.650, 95% CI: 0.143–2.961).

### Comparison of the Survival Outcomes Between the ARH and LRH Groups According to Different Stratifying Variables

**FIGURE 2:**
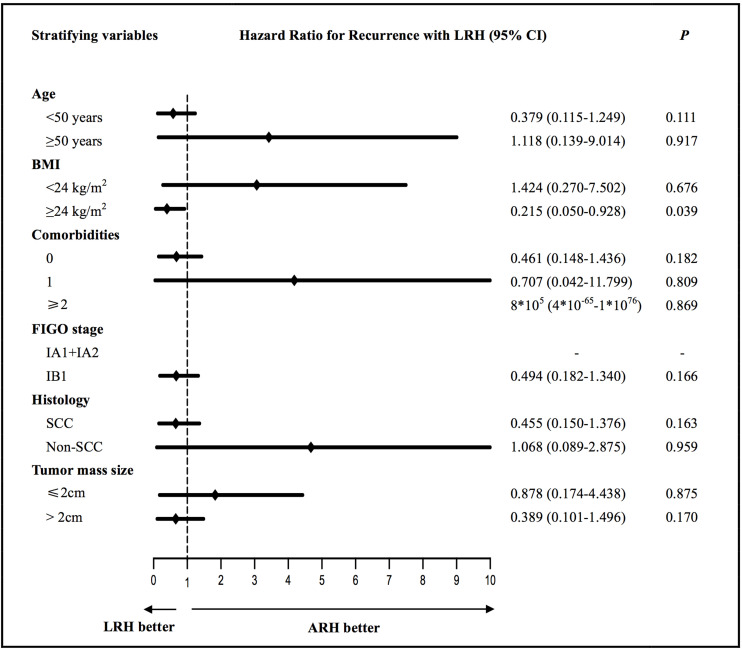
The multivariate subgroup analysis of the hazard ratios (black diamonds) and 95% CIs (horizontal lines) for the interactions between LRH and recurrences according to different stratifying variables. ARH, abdominal radical hysterectomy; LRH, laparoscopic radical hysterectomy; BMI, body mass index; SCC, squamous-cell carcinoma.

We evaluated the factors associated with PFS and OS that can be acquired preoperatively in the ARH and LRH groups according to different stratifying variables, which are shown in Supplementary Table S1. No differences were found in the PFS in the univariate analysis between the two groups according to the different stratifying subgroups except for the subgroup of patients with a BMI ≥ 24 kg/m^2^ (*P* = 0.040); the corresponding Kaplan-Meier survival curves are shown in Supplementary Figure S1A. Only three obese patients had BMI ≥ 30 kg/m^2^ according to the World Health Organization (WHO) standards, so the survival analysis did not make much sense. Thus, we further performed a subgroup analysis of obese patients who had BMI ≥ 28 kg/m^2^ according to the Chinese standard. However, no significant differences were found in the PFS (*P* = 0.140) and OS (*P* = 0.893) in patients with BMI ≥ 28 kg/m^2^ between the two surgery groups. Furthermore, no differences were found in the OS in the univariate analysis between the two groups according to the different stratifying subgroups. Then, survival outcomes were further estimated and characterized by different stratifying variables in the multivariate analysis. Figure 2 and Supplementary Figure S3 respectively show the HRs (black diamonds) and 95% CIs (horizontal lines) for the interactions between LRH and recurrences or death according to different stratifying subgroups in the multivariate subgroup analysis. This indicated that the operation approach was an independent factor of PFS in the subgroup of patients with a BMI ≥ 24 kg/m^2^ (*P* = 0.039, adjusted HR = 0.215, 95% CI: 0.050–0.928). Notably, no significant differences in PFS (*P* = 0.166, adjusted HR = 0.494, 95% CI: 0.182–1.340) and OS (*P* = 0.625, adjusted HR = 0.689, 95% CI: 0.155–3.065) were found for patients with FIGO stage IB1 between the ARH and LRH groups; the corresponding Kaplan–Meier survival curves are shown in Supplementary Figure S2. For patients with FIGO stage IA1 + IA2, none of them experienced recurrence or died.

### Comparison of the Survival Outcomes According to Different Periods of LRH Surgery Time

According to the year in which the laparoscopy procedures were performed, we divided all LRH cases into three groups: those performed during 2006–2012, 2013–2015, and 2016–2018. Each of these groups contained 21 (12.2%), 61 (35.5%), and 90 (52.3%) patients. The reason for dividing the patients this way was that we wanted to evaluate the laparoscopic skill of our team during the learning period and the stable period. Surgeons take 5–6 years to overcome the learning curve for laparoscopy (25). Furthermore, gynecologic oncologists might reach an acceptable level of surgical proficiency in LRH after approximately 20 cases and show a gentle slope of a learning curve (26). In Supplementary Table S2, the clinicopathological characteristics were roughly equal between these three groups, indicating that the survival analyses were comparable. However, the operation time of the 2006–2012 group was significantly longer than that of the 2013–2015 group, followed by the 2016–2018 group (201.9 vs. 188.2 vs. 163.3 min, *P* < 0.001). More cases in the 2006–2012 group than the other group required a blood transfusion (2 vs. 1 vs. 0, *P* = 0.011). The highest rate of postoperative complications was in the 2006–2012 group (14.3% vs. 23.0% vs. 7.8%, *P* = 0.031). No significant differences were observed in PFS (*P* = 0.126) and OS (*P* = 0.583) between the three groups in Supplementary Figure S4A,B. It was indicated that with the increasing number of cases, the laparoscopic skill of our team is becoming proficient and stable.

## Discussion

Since the first MIS was performed 100 years ago, the application of laparoscopy has become a general trend in the era of surgical treatment of tumors. In the management of early-stage cervical cancer, LRH yields benefits such as less blood loss, lower transfusion rates, shorter operative and hospitalization times, faster bowel and bladder function recovery, and fewer postoperative complications than the open approach (10–14). The advantages of LRH were also identified in our study and included shorter operative time, less operative blood loss, and a lower incidence of postoperative ileus.

However, the effectiveness of LRH for early-stage cervical cancer has been questioned since two high-quality research articles were published in the *New England Journal of Medicine* in 2018. The LACC trial found unexpected results that the PFS after MIS for cervical cancer ranging from IA1 disease with LVSI to IB1 disease was significantly worse than that after open surgery (3-year rate, 91.2% vs. 97.1%; 4.5-year rate, 86.0% vs. 96.5%), and the same was reported for the OS (3-year rate, 93.8% vs. 99.0%) (19). The retrospective study showed that increasing application of MIS in the United States was associated with a 0.8% decline per year in the 4-year relative survival rate of patients who underwent RH for cervical cancer from 2006 to 2010 (17). Moreover, recently, in a large multi-institutional retrospective study in the United States, the results showed that MIS was associated with worse PFS but not OS compared with the open approach, especially in patients with a tumor size ≤2 cm on final pathology (20). Nevertheless, some researchers are doubtful since the LACC trial recruited an average of only two patients per center per year for the MIS group (27, 28). Additionally, some surgeons who performed LRH for cervical cancer were simply gynecologists and might not have been experts in the field of gynecologic oncology. The criteria for the medical centers that recruited for the MIS group of the LACC trial were considered to lack validity in ensuring that the surgeons had sufficient experience (27, 29). The LACC trial may expose an important issue, namely, that the operative experience of the surgeon plays an important role in maximizing the survival benefits of laparoscopic surgery. Several authors have observed and described the learning curve for mastering the laparoscopy technique for RH and found that surgeons will become competent in performing LRH after 20–40 cases (26, 30, 31).

Therefore, to counteract the limitations of differing levels of laparoscopic skills in the LACC trial, we provided the results of a single gynecologic oncology team that performed RH over 18 years for the management of patients with early-stage cervical cancer. Laparoscopy was first introduced at PUMCH around 1980. After accumulating abundant successful experience with gynecological benign tumors for twenty years, laparoscopy was first applied to cervical cancer in 2006. Our team is composed of professional gynecologic oncologists, including three attending surgeons who can perform LRH independently, and several resident doctors and surgical assistants. Surgeons experienced in general surgery, hepatobiliary surgery and urologic surgery assisted in managing the complicated abdominal metastatic cases. The member of our team learned to perform ARH at first, then followed by LRH with accumulation of surgery cases. Thus, when facing the clearer surgical vision and anatomy during LRH, faster lymphadenectomies and more precise manipulation can be performed compared with ARH. The long learning curve and large number of cervical cancer cases guaranteed a stable and proficient level of laparoscopy techniques. As we know, the abdominal surgical incision of LRH is less invasive. In our study, the operation time of LRH was about one hour shorter than ARH (248.8 vs. 176.9 min), which was exactly the time to enter and closing abdominal cavity by the anatomical hierarchy in turn during ARH. In fact, we found no differences in the survival outcomes of LRH between patients in the early group and the late group. The rates of positive resection margins were low and identical between the LRH and ARH groups, which suggests that LRH did not result in inadequate surgical resection. Most importantly, the PFS and OS rates of the LRH group in our study were better than those in the LACC trial (3-year rate, 97.1% vs. 91.2% for PFS and 98.8% vs. 93.8% for OS). This evidence supports the idea that the surgeons in our study exhibited acceptable proficiency in performing LRH for the management of early cervical cancer.

As a result, we should consider whether the operation quality and survival outcome of patients who underwent LRH can be improved by culturing more mature laparoscopy skills of attending surgeons and by performing these procedures using a single experienced gynecology team. In contrast, some researchers have indicated that LRH has beneficial survival outcomes in select cervical cancer patients (32, 33). In our study, we explored whether the results would differ in groups of patients with unique features. Finally, we found that LRH is associated with better PFS in patients with a BMI greater than 24 kg/m^2^, which was in agreement with previous research (34). The reason for this can be explained in that overweight is associated with more postoperative complications by ARH, such as poor healing of the incision and a high rate of postoperative ileus. Thus, the results of the subgroup analysis may provide clues as to which operative approach should be chosen for the treatment of certain groups of patients. However, no significant differences were found in the survival outcomes for obese patients between the two surgery groups. The reason may be that the small number of obese patients in our study led to negative results.

In addition, early research has suggested potential factors that lead to an increase in recurrence rates with the laparoscopic approach. For example, abdominal insufflation with carbon dioxide is believed to foster tumor growth and peritoneal dissemination (35, 36). In our study, all included patients used uterine manipulators during LRH, and all colpotomies were performed intracorporeally in accordance with the traditional international consensus. However, after the LACC trial was published, we have considered the potential factors that have led to an increase in recurrence rates with the laparoscopic approach. The use of uterine manipulators squeezes the tumor mass, which results in tumor erosion and the spread of malignant cells in the abdominal cavity (37, 38). Furthermore, the patients are in the Trendelenburg position during MIS, and thus tumor cells easily collect in the upper abdomen and are more likely to implant there (18). In our research, recurrence outside the pelvis of the LRH group occurred more frequently as abdominal metastasis than that of the ARH group. To remedy the potential limitations of laparoscopic surgery described above, Yuan P. recently developed a modified LRH procedure with enclosed colpotomy. Instead of using a uterine manipulator, the uterus can be handled abdominally by the traction of the sutures on the round ligament, ovarian ligament and fallopian tube. The upper vagina is also ligated before resection of the upper third of the vagina to avoid exposing the tumor to the abdominopelvic cavity (39). Therefore, anticipating further improvements in the outcomes after LRH with modified techniques is reasonable (40).

The strength of our trial is that the data were collected for a single gynecologic oncology team, which guaranteed stable proficiency of the surgeons who performed LRH for the treatment of early cervical cancer. However, we further compared our ARH results with data reported by the LACC trial. The ARH group in our study had worse survival outcomes compared with those in the LACC trial (3-year rate, 89.3% vs. 97.1% for PFS and 95.2% vs. 99% for OS). The reason for this can be explained by the fact that our ARH patients were enrolled much earlier than those in the LACC trial. A decade ago, the medical imaging techniques were less advanced. Underestimation of tumor stage and relatively unimproved techniques for adjuvant therapy in the early years could explain the worse outcomes in our ARH group. Magnetic resonance imaging and positron emission tomography were demonstrated to be of higher sensitivity than computed tomography (41–43), but these modalities were not commonly used in China before 2010. With access to only a simple computed tomography scan at the time, gynecologic oncologists lacked detailed information for wider extension or metastasis of cervical cancer. Thus, the cervical cancer stage might have been underestimated before surgery, which may have reduced the comparability of the two cohorts in our study. Since the patients in our study underwent ARH mostly between 2000 and 2012, the ARH group included more high-risk patients than the LRH group, which might have negatively affected the survival outcomes. In addition, the techniques for radiotherapy have improved greatly over the last decade. Therefore, at an earlier time, adjuvant radiotherapy, especially radiotherapy, may not have had effects equivalent to the current methods. Furthermore, gynecologic oncologists in China may choose NACT for tumor downstaging, and subsequent RH is common to manage this group of patients, especially before 2010 (17–19). As we know, most of the retrospective studies were sequential comparisons rather than concurrent analyses. Thus, all the above factors may have contributed to the higher recurrence and short survival of the ARH group in our study compared with those in earlier studies. However, no significant differences were found in the clinicopathological characteristics between the two surgery groups, confirming that the variables were equally comparable for the survival analysis.

In addition to the main limitation, this study also has several other disadvantages. First, unknown potential confounders and selection biases may be present in this retrospective study due to the long period of data collection. However, we attempted to define patient inclusion criteria carefully to ensure all data were collected in a similar way and to always ensure uniformity between the two surgery groups. Moreover, we have balanced those confounding factors between the two groups by a Cox multivariate regression analysis when there were a few heterogeneities in baseline factors between the two groups. We also divided the dataset into homogenous subgroups and performed a stratification analysis. Second, we obtained data from only a single team, which resulted in a limited number of cases for the survival analysis. Nevertheless, we attempted to expand the range of patient data collection, which was over 18 years. For our single team, the number of cases with IA1-IIB was up to 368, which was comparably considerable. Finally, the median follow-up time of 59 months was slightly short, which may have led to a loss of some important outcomes for a small proportion of patients. However, the length of the follow-up time did not affect the results a lot in the statistics. In addition, we will continue to obtain more endpoints of the included cervical cancer patients and collect more information from new patients. Further prospective studies are needed to investigate the relationship between surgery type and survival outcomes. The aforementioned limitations are inherent characteristics of a single-team retrospective study. Although we have tried to reduce these limitations in various ways, they cannot be eliminated completely in a basic sense.

## Conclusion

In conclusion, this retrospective study shows that LRH had similar survival outcomes as ARH for stage IA1-IB1 cervical cancer patients. However, for patients with a BMI greater than 24 kg/m^2^, LRH is associated with better PFS than ARH. Additionally, LRH had more favorable surgical outcomes, including shorter operative time, less operative blood loss, and a lower rate of postoperative complications than ARH. Furthermore, the presence of more than two comorbidities was related to poor OS. The experience of our single team over 18 years demonstrated that LRH is not inferior to ARH for select cervical cancer patients treated by a single team with adequate laparoscopy experience. We look forward to collecting more information from cervical cancer patients in our center and learning from the research experience at other centers.

## Data Availability Statement

The raw data supporting the conclusions of this article will be made available by the authors, without undue reservation.

## Ethics Statement

The studies involving human participants were reviewed and approved by the Ethics Committee of Peking Union Medical College Hospital. The patients/participants provided their written informed consent to participate in this study.

## Author Contributions

L-YP and YJ: study concepts and manuscript review. MQ, L-YP, YJ, and H-FH: study design. MQ, YC, J-YC, and YL: data acquisition. YJ, WW, and YG: quality control of data and algorithms. MQ, LS, and Y-XW: data analysis and interpretation. MQ, LS, YS, and JY: statistical analysis. MQ and LS: manuscript preparation. All authors edited the manuscript.

## Conflict of Interest

The authors declare that the research was conducted in the absence of any commercial or financial relationships that could be construed as a potential conflict of interest.
