# Supplementary material for: A Comparison of Laparoscopies and Laparotomies for Radical Hysterectomy in Stage IA1-IB1 Cervical Cancer Patients: A Single Team With 18 Years of Experience
Source: Front Oncol. 2020 Aug 28;10:1738. doi: 10.3389/fonc.2020.01738 (PMC7485394; doi:10.3389/fonc.2020.01738)
Supplement: Supplementary file 1 [file Data_Sheet_1.docx]

**Supplementary materials**

**Supplementary Table**

**Table S1**. The univariate analysis of factors associated with PFS and OS in ARH and LRH group according to different stratifying variables.

| **Stratifying variables** | **N** | **PFS** |  | **OS** |
| --- | --- | --- | --- | --- |
|  |  | ***P*** |  | ***P*** |
| **Age (year)** |  |  |  |  |
| <50 | 196 | 0.099 |  | 0.350 |
| ≥50 | 60 | 0.874 |  | 0.310 |
| **BMI (kg/m^2^)** |  |  |  |  |
| <24 | 164 | 0.834 |  | 0.600 |
| ≥24 | 92 | 0.040 |  | 0.788 |
| ≥28 | 15 | 0.140 |  | 0.893 |
| **Comorbidities** |  |  |  |  |
| 0 | 213 | 0.087 |  | 0.140 |
| 1 | 34 | 0.383 |  | 0.602 |
| ≥2 | 9 | 0.408 |  | 0.502 |
| **FIGO stage** |  |  |  |  |
| IA1 (positive LVSI) +IA2 | 21 | - |  | - |
| IB1 | 235 | 0.182 |  | 0.835 |
| **Histology** |  |  |  |  |
| SCC | 204 | 0.135 |  | 0.623 |
| Non-SCC | 52 | 0.939 |  | 0.551 |
| **Tumor mass size** |  |  |  |  |
| ≤2 cm | 175 | 0.830 |  | 0.838 |
| >2 cm | 81 | 0.114 |  | 0.977 |

(Abbreviations: ARH, abdominal radical hysterectomy; LRH, laparoscopic radical hysterectomy; PFS, progression-free survival; OS, overall survival; BMI, body mass index; FIGO, International Federation of Gynecology and Obstetrics; SCC, squamous-cell carcinoma)

**Table S2.** The clinicopathological characteristic, surgical and survival outcomes of patients with LRH between 2006-2012, 2013-2015, and 2016-2018 in the LRH group.

| **Characteristics** | **Total**  **(N=172)** | **2006-2012**  **(N=21)** | **2013-2015**  **(N=61)** | **2016-2018**  **(N=90)** | ***P*** |
| --- | --- | --- | --- | --- | --- |
| **Clinical characteristics** |  |  |  |  |  |
| **Age (mean+SD)** | 44.3 (±8.2) | 46.3 (±5.7) | 42.8 (±8.4) | 44.9 (±8.5) | 0.937 |
| <50 year | 128 (74.4) | 15 (71.4) | 46 (75.4) | 67 (74.4) |  |
| ≥50 year | 44 (25.6) | 6 (28.6) | 15 (24.6) | 23 (25.6) |  |
| **BMI (kg/m^2^)** | 23.1 (±2.8) | 22.4 (±2.5) | 23.2 (±2.8) | 23.2 (±2.9) | 0.238 |
| <24.0 | 112 (65.1) | 17 (81.0) | 37 (60.7) | 58 (64.4) |  |
| ≥24.0 | 60 (34.9) | 4 (19.0) | 24 (39.3) | 32 (35.6) |  |
| **Comorbidities** |  |  |  |  | 0.103 |
| 0 | 139 (80.8) | 20 (95.2) | 44 (72.1) | 75 (83.3) |  |
| 1 | 26 (15.1) | 1 (4.8) | 12 (19.7) | 13 (14.4) |  |
| ≥2 | 7 (4.1) | 0 | 5 (8.2) | 2 (2.2) |  |
| **FIGO stage** |  |  |  |  | 0.346 |
| IA1 with positive LVSI | 3 (1.7) | 1 (4.8) | 0 | 2 (2.2) |  |
| IA2 | 14 (8.1) | 3 (14.3) | 3 (4.9) | 8 (8.9) |  |
| IB1 | 155 (90.2) | 17 (81.0) | 58 (95.1) | 80 (88.9) |  |
| **Histology** |  |  |  |  | 0.335 |
| Squamous-cell carcinoma | 132 (76.8) | 19 (90.5) | 49 (80.3) | 64 (71.1) |  |
| Adenocarcinoma | 35 (20.3) | 2 (9.5) | 10 (16.4) | 23 (25.6) |  |
| Adensquamous carcinoma | 5 (2.9) | 0 | 2 (3.3) | 3 (3.3) |  |
| **Tumor grade** |  |  |  |  | 0.708 |
| G1 | 97 (56.4) | 10 (47.6) | 38 (62.3) | 49 (54.4) |  |
| G2 | 43 (25.0) | 6 (28.6) | 12 (19.7) | 25 (27.8) |  |
| G3 | 32 (18.6) | 5 (23.8) | 11 (18.0) | 16 (17.8) |  |
| **Adjuvant treatment** | 64 (37.2) | 4 (19.0) | 28 (45.9) | 32 (35.6) | 0.036 |
| **Surgical and survival outcomes** |  |  |  |  |  |
| **Length of operation time (minute)** | 176.9 (±38.3) | 201.9 (±48.3) | 188.2 (±30.2) | 163.3 (±35.3) | <0.001 |
| **Volume of intraoperative blood loss (ml)** | 200.9 (±121.5) | 263.3 (±201.6) | 211.8 (±122.3) | 178.9 (±87.4) | 0.136 |
| **The presence of blood transfusion** | 3 (1.7) | 2 (9.5) | 1 (1.6) | 0 | 0.011 |
| **Postoperative complications** |  |  |  |  |  |
| No | 148 (86.0) | 18 (85.7) | 47 (77.0) | 83 (92.2) | 0.031 |
| Infections | 10 (5.8) | 1 (4.8) | 7 (11.5) | 2 (2.2) | 0.057 |
| Ureteral fistula | 3 (1.7) | 1 (4.8) | 2 (3.3) | 0 | 0.169 |
| Urinary dysfunction | 8 (4.7) | 1 (4.8) | 6 (9.8) | 1 (1.1) | 0.044 |
| Ileus | 0 (0) | 0 | 0 | 0 |  |
| Lymphocele | 5 (2.9) | 0 | 2 (3.3) | 3 (3.3) | 0.699 |
| Thrombus | 3 (1.7) | 0 | 2 (3.3) | 1 (1.1) | 0.491 |
| Stay in ICU | 1 (0.6) | 0 | 0 | 1 (1.1) | 0.632 |
| **No recurrence** | 164 (95.3) | 20 (95.2) | 55 (90.2) | 89 (98.9) |  |
| **Recurrent death** | 4 (2.3) | 0 | 3 (4.9) | 1 (1.1) |  |
| **Recurrent but alive** | 4 (2.3) | 1 (4.8) | 3 (4.9) | 0 |  |

Data are presented as number (%) or mean (±SD) or median (±IQR). (Abbreviations: BMI, body mass index; FIGO, International Federation of Gynecology and Obstetrics; ICU, Intensive care unit)

**Supplementary Figure**

| **** |
| --- |

**Figure S1.** The Kaplan–Meier curves of PFS with BMI≥24 kg/m^2^ (A) and patients with BMI<24 kg/m^2^ (B) between ARH and LRH groups. (Abbreviations: ARH, abdominal radical hysterectomy; LRH, laparoscopic radical hysterectomy; PFS, progression-free survival)

**B**

**A**

**Figure S2.** The Kaplan–Meier curves of PFS (A) and OS (B) for patients with FIGO stage IB1 between ARH and LRH groups.

(Abbreviations: ARH, abdominal radical hysterectomy; LRH, laparoscopic radical hysterectomy; PFS, progression-free survival; OS, overall survival;)

| **Subgroup** | **Hazard Ratio for Death with LRH (95% CI)** | | ***P*** |
| --- | --- | --- | --- |
| **Age** |  |  |  |
| <50 years |  | 0.397 (0.065-2.413) | 0.316 |
| ≥50 years |  | 1*10^4^(1*10^-203^1*10^211^) | 0.969 |
| **BMI** |  |  |  |
| <24 kg/m^2^ |  | 0.551 (0.074-4.097) | 0.560 |
| ≥24 kg/m^2^ |  | 0.758 (0.071-8.137) | 0.819 |
| **Comorbidities** |  |  |  |
| 0 |  | 0.258 (0.028-2.416) | 0.235 |
| 1 |  | 14. 2 (1*10^-53^-2*10^55^) | 0.967 |
| ≥2 |  | 4*10^4^ (2*10^-16^-7*10^24^) | 0.658 |
| **FIGO stage** |  |  |  |
| IA1+IA2 |  | - | - |
| IB1 |  | 0.689 (0.155-3.065) | 0.625 |
| **Histology** |  |  |  |
| SCC |  | 0.578 (0.117-2.859) | 0.501 |
| Non-SCC |  | 14.1 (10*10^-28^-2*10^29^) | 0.936 |
| **Tumor mass size** |  |  |  |
| ≤2cm |  | 0.565 (0.067-4.746) | 0.599 |
| > 2cm |  | 0.824 (0.086-7.847) | 0.866 |
|  |  |  |  |
|  |  |  |  |
| **ARH better**  **LRH better** | | |  |

**Figure S3.** The multivariate subgroup analysis of the hazard ratios (black diamonds) and 95 % CIs (horizontal lines) for the interactions between LRH and death according to different stratifying variables.

(Abbreviations: ARH, abdominal radical hysterectomy; LRH, laparoscopic radical hysterectomy; BMI, body mass index; SCC, squamous-cell carcinoma)

|   **B**  **A** |
| --- |
|  |

**Figure S4.** The Kaplan–Meier curves of progression-free survival (A) and overall survival (B) according to different periods of LRH surgery time. There were similar PFS (A) and OS (B) in patients with LRH between 2006-2012, 2013-2015, and 2016-2018 groups. (Abbreviations: LRH, laparoscopic radical hysterectomy; PFS, progression-free survival; OS, overall survival.)
